# Development and validation of a Semi-quantitative food frequency questionnaire among older people in north of Iran

**DOI:** 10.22088/cjim.9.1.78

**Published:** 2018

**Authors:** Ali Bijani, Haleh Esmaili, Reza Ghadimi, Atekeh Babazadeh, Reyhaneh Rezaei, Robert G Cumming, Seyed Reza Hosseini

**Affiliations:** 1 Social Determinants of Health Research Center, Health Research Institute, Babol University of Medical Sciences, Babol, Iran; 2 Mobility Impairment Research Center, Health Research Institute, Babol University of Medical Sciences, Babol, Iran; 3 3.Cancer Research Center, Health Research Institute, Babol University of Medical Sciences, Babol, Iran; 4 School of Public Health, University of Sydney, Sydney, Australia

**Keywords:** Reliability, Semi quantitative food frequency questionnaire, Cohort study, Elderly.

## Abstract

**Background:**

The study was conducted to assess reliability of modified semi-quantitative food frequency questionnaire (SQFFQ) as a part of the Amirkola Health and Aging Project (AHAP).

**Methods:**

The study was carried out in a sample of 200 men and women aged 60 years and older. A 138-item SQFFQ and two 24-hour dietary recalls were completed. The reliability of SQFFQ was evaluated by comparing eighteen food groups, energy and nutrient intakes derived from both methods using Spearman and Pearson’s correlation coefficients for food groups and nutrients, respectively. Bland-Altman plots and Pitman’s tests were applied to compare the two dietary assessment methods.

**Results:**

The mean (SD) age of subjects was 68.16 (6.56) years. The average energy intake from 24-hour dietary recalls and the SQFFQ were 1470.2 and 1535.4 kcal/day, respectively. Spearman correlation coefficients, comparing food groups intake based on two dietary assessment methods ranged from 0.25 (meat) to 0.62 (tea and coffee) in men and from 0.39 (whole grains) to 0.60 (sugars) in women. Pearson correlation coefficients for energy and macronutrients were 0.53 for energy to 0.21 for zinc in male and 0.71 for energy to 0.26 for vitamin C in females. The Pitman’s test reflected the reasonable agreement between the mean energy and macronutrients of the SQFFQ and 24-hour recalls.

**Conclusions:**

The modified SQFFQ that was designed for the AHAP was found to be reliable for assessing the intake of several food groups, energy, micro-and macronutrients.

Dietary intake is a crucial modifiable risk factor for many diseases and also has an important role in the management of chronic diseases (1-3). Therefore the accuracy and reliability of information related to people’s diet is fundamental for examining and monitoring nutritional status, identifying dietary risk factors and food insecurity (4, 5). Among the various methods that assess dietary intake, the food frequency questionnaire (FFQ) has been proposed as the most common and acceptable dietary instrument to obtain the usual and long term food intake in epidemiological studies among all age groups (6, 7). However, FFQ only collects information on the frequency of food consumption, and so a better dietary assessment method is the semi-quantitative food frequency questionnaire (SQFFQ) that relies on questions about frequency and portion sizes (8). Although, the SQFFQ has own weaknesses, its strength and advantages include being easy to use, inexpensive and reflecting usual long term (8). One of the main weaknesses of SQFFQ is that the standard version is not usable in different parts of the world, even for different regions of a country, because of food diversity, variation of food habits and food choices in different geographic areas, ethnic groups and cultures (9). So, it is necessary to develop, modify and adapt the questionnaire to cover and reflect individual’s usual food intake in particular regions.

Hence, evaluation of a modified SQFFQ’s is the foundation of community and epidemiological nutrition studies (10-13). Dietary assessment among older people can be particularly difficult because of fading memory, attention disorder and difficulty to recalling food portion sizes (14, 15). We developed and adapted a validated SQFFQ for using in a cohort study of older people in the North of Iran (16). The aim of this study described in this paper was to develop modified SQFFQ that accounts for usual foods in Northern Iran and to compare the findings of the SQFFQ with the data from two 24-hour dietary recalls. The study was done as a part of the Amirkola Health and Aging Project (AHAP).

## Methods

**Study design and population:** The subjects of the present study were a part of the AHAP cohort study, conducted in Amirkola, Mazandaran, in the North of Iran (17, 18). In this study, 15 out of 215 elders were excluded because they did not complete SQFFQ or/and two 24-hour dietary recalls. Therefore, the current study was carried out between December 2016 and March 2017 on 200 community dwelling older adult participants, aged 60 years and older by simple random sampling from the AHAP cohort.

**Dietary assessment: **The SQFFQ that was developed for this study was modified from a Willet format FFQ for Iranian populations that was validated for people who live in Tehran (19, 20). Initially, the SQFFQ included 168 single national food items based on the most frequent food items eaten by older people in the study area as reported by experienced nutritionists familiar with the local diet. For instance, turkey, duck and goose are commonly consumed by local elders and so were added in the questionnaire. The frequency of consumption and serving size was recorded on a daily, weekly and monthly basis. In the second step, to prevent elders’ hesitation and boredom, the number of food items were reduced to 138 by aggregating some similar foods which share both nutritional content and serving, for example mixed vegetables that are added to rice, stuffed chicken or fish and some other Iranian dishes. Because of subjects’ limitation of knowledge of food portions and conceptualization skills, the food interview was conducted by trained interviewers in the study center. Furthermore, to obtain accurate information, some portion sizes (for example, rice, milk, fruit juice, tomato paste) were illustrated with pictures or by using household measures (glass, plate, spoon, and bowl) and others were recorded in natural units as small, medium or large size (for example fruits, tomato, cucumber). For assessing the reliability of SQFFQ, two 24-hour recalls were used as references for each participant. This questionnaire was done face-to- face and recorded all foods and beverages consumed in the previous day from the time of waking in the morning to going to sleep at night. Detailed information about food recipes, preparation methods and ingredients, quantity and venue of food intake were considered. 

In addition, because of the diverse methods used to prepare mixed dishes, the exact ingredients were recorded according to subjects’ reports; for instance, some stews have different ingredients according to families’ food habits. To improve precision of estimation, for the subjects (men and/or women) with poor memory or inability to prepare their own food, we asked a person who lived with them, and was aware from their dietary intake and/or prepared their food to participate in the interview.

**Food analysis:** The reported frequency for each food item in the SQFFQ was converted to gram per day according to measuerment of household Iranian foods (21). The weight of seasonal foods, especially some fruits and vegetables (for instance, citrus fruits, peach, pear, and pumpkin) was calculated based on the number of seasons or months during which they were available. For each person, data from the SQFFQ and the mean intake of from the two 24-hour recalls were entered into Nutritionist Software Version IV to calculate the daily energy and nutrient intakes. In addition, the food items on the SQFF and 24-h recalls were categorized into 18 food groups based on their similarities in nutrient contents (table 1) (22, 23).

**Statistical analysis:** Data were entered into SPSS Version 21 and STATA Version 12. The Kolmogorov–Smirnov test was used to assess the normality of mean food and nutrient intakes. Differences between the SQFFQ and the mean of the two 24-hour recalls were obtained using the Wilcoxon test for food groups and paired t-test for macro and micro nutrients. 

In addition for analysis, the Spearman and Pearson’s correlation were calculated to determine the correlation between the amounts of food groups and nutrient intakes as determined by each method. Additionally, Bland-Altman plots and Pitman’s tests were applied to compare the two dietary assessment methods. P-values less than 0.05 were considered statistically significant.

**Table 1:** Food groups used in the study of reliability of the food frequency questionnaire developed for the AHAP

**Food groups** **(n=18)**	**Food items ** **(n=138)**
Whole grains	Barbari, Sangak, oat, dark toasted bread
Refined grains	Taftoon, Lavash, rice, white toasted bread,Baguette
Dairy products	High and low fat dairies (milk, yogurt, cheese, curd, dough) and ice cream
Vegetables	Raw and cooked, lettuce, celery, green pea, spinach, mushroom, tomato, cucumber, squash, eggplant, carrot, garlic, onion, green pepper, turnip, green chilies green bean, Cruciferous vegetables Pumpkin, mixed vegetable (used in Persian cuisine).
Fruits	Pear, apricot, apple, cherry, peach, nectarine, green plum, fig, grapes, kiwi, grapefruit, orange, tangerine, persimmon, tangerine, pomegranate, dates, cherry, prune, sour cherry, strawberry, banana, sweet lemon, lime lemon, mulberry,Cantaloupe, Persian melon, watermelon dried fruits, fresh juice.
Legumes	Red, white, kidney, black eye beans, chickpea, broad bean, soy bean, split bean, mung and lentil.
Nuts and seeds	Cashew, almond, pistachio, peanut, hazelnut, sesame, pumpkin and sunflower seeds
Solid fat	Hydrogenate oil, butter, margarine, animal oil and tallow
Liquid oil	Vegetable oil (canola, olive, sunflower, ets.)
Meats	Red meat (Lamb, veal, beef), ground meat, organ meat (brain, tongue, feet, tripe and head, liver, kidney, and heart), sausage, hamburger.
Poultry	Chicken, geese, turkey, duck, rooster and their organs (gizzard, heart and liver)
Fish	All kinds of fish (fresh, freeze and canned)
Egg	Egg (all preparation)
Soft drinks	All soft and sweet drinks, non-alcoholic beer, syrup and canned fruit juices
Sugar	White and brown sugar, candy, noghl
Honey and jam	Honey and all kind of jam
Snack and dessert	All kinds of cake, muffins, chips, chocolates, pastries (non-cream and creamy), all biscuits, gaz, sohan, popcorn, cheese puffs
Tea and coffee	All kinds of tea (green, red, white), coffee

## Results

A total of 200 older people (100 men and 100 women) completed the SQFFQ and two 24-hour recalls. Selected characteristics of study participants are shown in table 2. The mean (SD) age of subjects was 68.16 (6.56) years. The mean (SD) energy intake from the two 24-hour dietary recalls was 1470.2 (481.2) kcal/day, and from the SQFFQ, it was 1535.4 (473) kcal/day, which was significantly higher (p=0.01). The daily average intake of 18 food groups, according to gender, as measured by at least two 24-hour dietary recalls and by the SQFFQ are reported in table 3. The medians on the SQFFQ were significantly higher for whole grains, dairy products, vegetables, fruits, sugar, egg and tea and coffee for both genders (p<0.05), while poultry was significantly underestimated by the SQFFQ in both males and females (p<0.05). A significant difference in liquid oil and soft drink was observed only in men, whereas snack and dessert intake was significantly different only in women.

**Table 2:** Characteristics of study population (200 subjects

**Characteristics**	**n (%)**
**Gender**	
Male Female	100(50) 100(50)
**Marital Status**	
Married Single	179(89.5) 21(10.5)
**Occupation**	
Housewife Farmer Labor Business Retired Unemployed	92(46) 22(11) 2(1) 29(14.5) 47(23.5) 8(4)
**Education**	
Uneducated Secondary school High school and higher	119(59.5) 50(25) 31(15.5)

* single includes unmarried, widow and divorced

**Figure 1:**
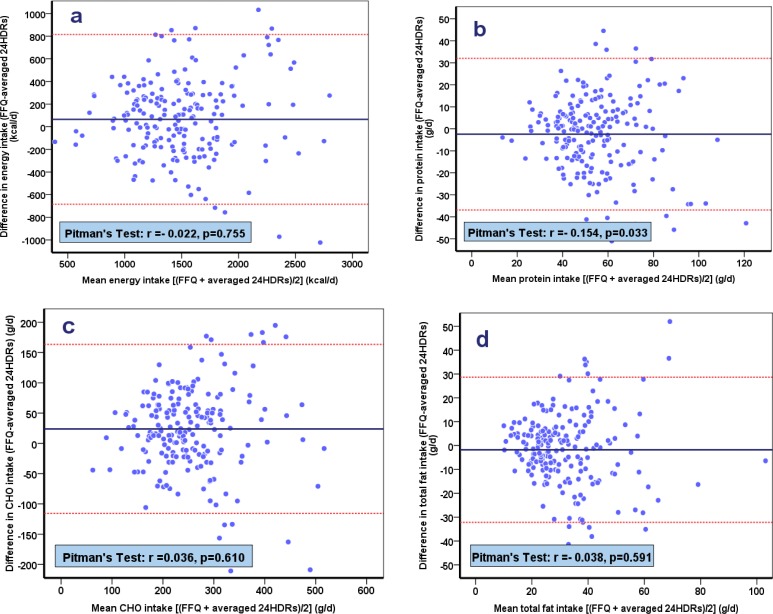
Bland-Altman plots for agreement between the SQFFQ and the average of two 24-hour recalls for(a) energy, (b) protein, (c) carbohydrate, and (d) fat intake

Spearman correlation coefficients were high and statistically significant for whole grains, nuts, liquid oil, sugar and tea among males and for refined grains in women (r>0.4, p<0.05). Dairy products, sugar, tea and coffee and solid oil were significantly correlated in both males and females (r>0.4, p<0.05). Table 4 shows the mean values and correlation coefficients for energy, macro-and micro nutrients for the mean of two 24-hour recalls and the SQFFQ. Mean carbohydrate intakes for both genders, as estimated by the SQFFQ were significantly higher than the intake estimated by the 24-hour and recalls the SQFFQ. Intake of total fat in males was significantly lower by the SQFFQ (table 4). The mean intake of most micronutrients was not significantly different between two dietary assessment methods, with the exception of manganese (table 4). Energy, protein, carbohydrate and total fat by the SQFFQ were statistically significantly correlated with intakes on the 24-hour dietary recalls. In addition, of the eight vitamins assessed, six showed significant correlations (vitamins A, B1, B2, B3, C and A- tocopherol) and of nine minerals (K, Ca, P, Se, Fe, Zn, Mg, Mn, and Cu), only Fe was not statistically significantly correlated between methods. In males, correlation coefficients were high for energy (r= 0.53) and carbohydrate (r= 0.52) and low for vitamin A (r=0.22) and Zn (r=0.21). In females, correlation coefficients were high for energy (r=0.71), carbohydrate (0.69) and vitamin B1(r= 0.67) and low for dietary fiber (r= 0.19) and vitamin C (r= 0.26). Generally, correlation coefficients were higher in females than in males, except for dietary fiber and manganese. Figure 1 shows the Bland–Altman plots for total energy, protein, carbohydrate and fat intake estimated from the SQFFQ and from the 24-hour recalls. Limits of agreement for total energy were between –684.236 to 814.753, for total protein intake between -36.902 to 32.039,for carbohydrate between –115.756 to 163.363 2.19 and for fat between -32.190 to 28.664. In the plots, the spread around the mean for energy, carbohydrates, protein and fat spread show consistent variations across all levels of intake and only a few participants fell outside the limit of agreements. For all measurements, the mean differences were not associated with the means of the two methods, confirming an acceptable and level of agreement (figure 1).

**Table 3:** Median intake and correlation coefficient for 18 food groups measured by SQFFQ and mean of two 24-h dietary recalls, according to gender

**Food groups ** **(g)**	** Male**			** Female**		
	**SQFFQ** **Median(IQR)**	**24-h recall** **Median(IQR)**	**r**	**SQFFQ** **Median(IQR)**	**24-h recall** **Median(IQR)**	**r**
Whole grains	195(141.0-312.2) ‡‡‡	177.1(107.2-240.9)	0.44***	141.0(94.0-188.0) ‡‡‡	108.4(58.8-152.8)	0.39***
Refined grains	264.7(250.0-400.0)	284.6(215-395)	0.33**	250.0(119.0-262.3)	193.8(119.1-266.7)	0.41***
Dairy products	150(83.0- 265.9) ‡‡	121.3(30.0-254.4)	0.43***	122.5(45.9-240.5) ‡‡‡	72.5(18.0-148.2)	0.48***
Vegetables	203.7(138.7-270.1) ‡	166.6(65.3-258.8)	0.33***	184.0(124.0-248.8) ‡	146.6(72.6-234.9)	0.36***
Fruits	283.6(222.9-434.2) ‡‡	227.4(110.8-390.4)	0.25*	273.6(172.7-390.4) ‡‡	204.6(97.9-327.3)	0.35***
Legumes	18.1(8.8-37.9)	0.0 (0.0-45.9)	0.06	12.8(4.0-29.4)	0.0(0.0-30.2)	0.15
Nuts and seeds	1.2(0.0- 6.0)	0.0 (0.0-6.0)	0.40***	1.4(0.0-6.0)	0.0(0.0-5.0)	0.36
Solid fat	0.7(0.0-3.9)	0.0(0.0-4.8)	0.50***	0.6(0.0-5.4)	0.0(0.0-7.0)	0.53***
Liquid oil	4.8(0.3-6.0) ‡	4.5(1.1-10)	0.40***	3.0(0.0-6.0)	3.0(0.5-7.5)	0.21*
Meat	8.8(3.8-18.0)	0.0 (0.0-32.5)	0.25*	4.9(1.6-9.3)	0.0(0.0-13.5)	0.13
Poultry	20(10.2-30.0) ‡‡	30.2(1.1-56.1)	0.18	14.5(7.9-27.3) ‡‡	27.0(0.5-42.8)	0.25**
Fish	5.3(2.7-12.7)	0.0 (0.0-40.0)	0.06	4.0(1.3-6.7)	0.0(0.0-14.6)	0.15
Egg	21.9(12.4-34.9) ‡	3.6(0.0-27.5)	0.17	13.2(7.5-23.6) ‡‡	0.0(0.0-18.0)	-0.01
Soft drinks	0.0 (0.0-9.3)	0.0 (0.0-0.0)	0.10	0.0(0.0-0.0)	0.0(0.0-0.0)	0.14
Sugar	11.8(2.0-21.4)	9.8(2.8-15.0)	0.42***	5.5(0.7-13.1) ‡‡‡	4.0(0.0-9.0)	0.60***
Honey and jam	2.4(0.0-9.6)	0.0(0.0-10.0)	0.38***	2.2(0.0-8.5)	0.0(0.0-5.0)	0.25*
snacks	1.0(0.0-6.2)	0.0(0.0-7.1)	0.07	0.0(0.0-5.0) ‡	0.0(0.0-12.4)	0.16
Tea and coffee	600(360-1080) ‡‡	480.0(315.0-720.0)	0.62***	540.0(480.0-720.4) ‡‡‡	450.0(335.0-600.0)	0.53***

IQR: interquartile range, r: spearman’s rho. Wilcoxon test (SQFFQ vs. 24 dietary recalls):

‡ p< 0.05;

‡‡ p<0.01,

‡‡‡ p<0.001 and in Spearman correlation:

* p<0.05;

** p<0.01;

*** P < 0.001was considered as significance

**Table 4.** Mean intake and correlation coefficient for nutrients were measured by SQFFQ and mean of two 24-h dietary recalls according to gender

**Nutrients **	** Male**			** Female**		
	**SQFFQ** **Mean(SD)**	**24-h recall** **Mean(SD)**	**r**	**SQFFQ** **Mean(SD)**	**24-h recall** **Mean(SD)**	**r**
Energy (kcal)	1760.5(487.6)	1715.9(489.7)	0.53***	1310.4(332.8) ‡‡	1224.4(322.1)	0.71***
CHO (g)	308.8(90.7)‡‡	283.8(89.7)	0.52***	224.5(60.3) ‡‡‡	201.9(56.7)	0.69***
Protein (g)	62.04(18.0)	65.9(20.1)	0.39***	44.58(12.0)	45.4(13.5)	0.55***
Total fat(g)	33.6(15.8) ‡	37.2(16.4)	0.49***	28.4(13.7)	28.3(13.2)	0.46***
SFA(g)	9.7(4.3)	10.4(5.3)	0.44***	8.2(3.6)	7.8(3.9)	0.46***
MUFA(g)	9.9(6.7) ‡‡‡	10.6(6.0)	0.24**	8.2(5.0)	7.7(4.1)	0.11
PUFA(g)	7.9(4.8)	9.9(5.8)	-0.10	7.2(5.6)	8.0(5.5)	-0.01
Cholesterol(mg)	185.6(91.9)	187.5(130.3)	0.25**	63.5(6.3)	114.4(80.3)	0.13
Dietary fiber (g)	15.7(7.8)	14.6(7.2)	0.25**	12.1(4.3)	12.1(4.9)	0.19*
Calcium (mg)	668.0(249.0)‡	595.8(243.8)	0.25*	504.5(205.1)	435.5(184.7)	0.41***
Phosphor(mg)	719.2(320.1)	754.4(331.1)	0.34***	543.7(214.4)	537.7(185.0)	0.55***
Potassium(mg)	2159.5(980.8)	2109.0(797.8)	0.28***	1771.3(636.1)	1752.1(592.5)	0.51***
Magnesium(mg)	191.7(126.2)	180.9(74.6)	0.36**	147.4(56.6)	144.0(46.9)	0.55***
Iron (mg)	12.7(5.0)	13.9(5.9)	0.15	9.7(4.4)	10.3(6.2)	0.10
Zinc (mg)	5.5(2.4)	5.9(2.6)	0.21*	4.1(1.5)	4.1(14)	0.31**
Selenium(mg)	0.08(0.03)	0.08(0.03)	0.29**	0.06(0.03)	0.06(0.03)	0.49***
Cupper(mg)	0.98(0.55)	1.02(0.48)	0.29**	0.76(0.28)	0.79(0.28)	0.31**
Manganese (mg)	3.05(1.52)‡	2.71(0.93)	0.46***	2.6(1.1)‡‡‡	2.22(0.79)	0.31**
Vitamin A	493.7(272.7)	454.6(374.7)	0.22*	428.2(239.0)	374.9(307.6)	0.16
Vitamin B1(mg)	2.05(0.58)	1.94(0.60)	0.45***	1.46(0.4)‡	1.33(0.3)	0.67***
Vitamin B2(mg)	1.24(0.45)	1.16(0.41)	0.34***	0.95(0.3)‡	0.85(0.2)	0.57***
Vitamin B3(mg)	17.5(5.4)‡	18.9(6.1)	0.30**	12.4(3.5)‡	13.3(4.8)	0.46***
Vitamin C (mg)	133.2(77.3)‡	111.9(86.5)	0.26**	107.8(56.1)	106.2(73.6)	0.26**
Vitamin E	3.28(2.24)	2.89(2.02)	-0.38	2.64(1.67) ‡	2.20(1.3)	0.05
Folate (mg)	229.7(113.4)	214.5(139.6)	0.16	187.8(109.7)	177.4(100.3)	0.17
Vitamin B12 (mg)	1.9(1.3)	2.2(1.5)	0.10	1.3(0.6)	1.3(0.9)	0.40***
Vitamin B6(mg)	0.9(0.3)	0.9(0.3)	0.09	0.7(0.2)‡‡	0.8(0.3)	0.34***
α-Tocopherol (mg)	3.9(2.6)	4.1(2.4)	0.27**	3.3(1.8)	3.3(2.2)	0.32**

CHO (carbohydrate), SFA (saturated fatty acid), MUFA (mono unsaturated fatty acid), PUFA (poly unsaturated fatty acid).r: Pearson’s correlation. Mean difference Paired t-test:

‡ p< 0.05;

‡‡ p<0.01,

‡‡‡ p<0.001and in Pearson correlation:

* p<0.05;

** p<0.01;

*** p<0.001was considered as significance.

## Discussion

In the present study, we assessed the reliability of a 138-item SQFFQ adapted for dietary intake of older people in the North of Iran. The participants were a sub-sample of the cohort study of Amirkola Health and Aging Project (AHAP) population. Since, the 24-h dietary recall has a better quality response, and illustrates the normal food choice and habits of population rather precisely (24), in the current study the 2-day dietary recalls were selected and applied as the reference to assess reliability of relative validated SQFFQ. We found relatively good correlation (r>0.4) between SQFFQ and dietary recalls for most food groups in both genders. The higher correlations for dairy products, solid oils, sugar and tea may be due to high intakes of these food items in this region. The results showed overestimation of some food groups using the SQFFQ compared with 24-hour dietary recalls. These over and under estimation of food groups can be related to the social beliefs on healthy and unhealthy foods and diet (22).

Additionally, frequent odontostomatological and masticatory problems are the other reasons that can decrease some food consumption like high fiber vegetables, whole grains or nuts. Also, the problems of exact amount of food record during the last year can lead to weak correlation in food groups between two methods. Furthermore, change of elderlies’ appetite in different days due to effect of medicine, mental and physical health status-which is common among them- and also seasonal dietary intakes, may cause lower correlations between the dietary assessment methods. The difference between correlation coefficients of food items in our study and other studies is related to different in food habits, dietary patterns and agriculture and food availability in this part (23, 25-27). In the current study, acceptable correlations were observed between our SQFFQ compared to the average of two 24-hourdietary recalls, with the exception of folate, B6, B12 and Fe. The instability of physical health status, accessible or/and affordable issues to provide nutrient resources can be reasons of insignificant correlation of these nutrients between two questionnaires. The present study found relatively correlation coefficients for most micronutrients (r> 0·4) (28), especially for elderlies that have some degrees of cognitive problems compared with young adults (14, 24, 29). The values of correlation coefficients were not the same between both genders for several nutrients, This may be related to the variations of portion size and frequency of food items consumed by males and females (30). Generally, unlike other studies the SQFFQ of our study did not overestimate nutrient intake (24, 29). Moreover, the different reference methods to collect dietary information like the number of days that dietary recalls recorded and the number of SQFFQs is an another reason of discrepancy in correlation coefficients between nutrients and food groups in different studies (22). In the current study, the Bland-Altman plots shows good agreement between methods for intakes of energy and macronutrients, similarly shown in some other studies even though a few studies identified inverse results on adults and old ages (31-35).

To our knowledge, this study is the second study (the first study was done in Golestan province) from the North of Iran which has investigated the reliability of SQFFQ. The reliability of SQFFQ for nutrients and food groups has been done before among adults in Tehran and Golestan*(22, 24, 29), but in our study Amirkola is the first to evaluate SQFFQ among the older people. Our study had several strengths. First, we asked participants to come to the diet interview with a person who lived with them, aware of their dietary intakes and/or who prepared their meals. Second, this study takes into consideration the frequency and the amount of each food group eaten by older people with the ability to separate values by gender. Errors in dietary assessment in studies of older population can be due to ability to estimate usual frequency food intake and portion size and fatigue because of lengthy questionnaire (35, 36). Therefore, we simplified the SQFFQ and used pictures and household measurements to better estimate the portion size.

A limitation of our study was the relatively the small sample size. Moreover, the study used only one SQFFQ and two 24-hour dietary recalls which are not sufficient to prevent the daily and seasonal food differences in our population. In conclusion, the SQFFQ developed for the elderly population in AHAP is acceptable for this population. Using a combination of nutrients and food groups, our SQFFQ is sufficiently reliable to be used to estimate dietary intake of older people in the North with reasonable reliability, agreement and correlation.
